# Crystal structure and Hirshfeld surface analysis of *N*-{2-[(*E*)-(4-methyl­benzyl­idene)amino]­phen­yl}-2-(5-methyl-1-*H*-pyrazol-3-yl)acetamide hemihydrate

**DOI:** 10.1107/S2056989018017747

**Published:** 2019-01-08

**Authors:** Karim Chkirate, Sevgi Kansiz, Khalid Karrouchi, Joel T. Mague, Necmi Dege, El Mokhtar Essassi

**Affiliations:** a Laboratory of Heterocyclic Organic Chemistry URAC 21, Pole of Competence Pharmacochemistry, Ave. Ibn Battouta, BP 1014, Faculty of Sciences, Mohammed V University, Rabat, Morocco; b Ondokuz Mayıs University, Faculty of Arts and Sciences, Department of Physics, 55139, Samsun, Turkey; c Physicochemical Service, Drugs Quality Control Laboratory, Division of Drugs and Pharmacy, Ministry of Health, 10100 Rabat, Morocco; d Department of Chemistry, Tulane University, New Orleans, LA 70118, USA

**Keywords:** crystal structure, pyrazole, hydrogen bonding, N—H⋯π inter­actions, C—H⋯π(ring) inter­actions, Hirshfeld surface analysis

## Abstract

The asymmetric unit of the title compound, C_20_H_20_N_4_O·0.5H_2_O, contains two independent organic mol­ecules (1 and 2) and a water mol­ecule of crystallization. The two mol­ecules differ primarily in the dihedral angles between the aromatic rings, which are 7.79 (7) and 29.89 (7)° in mol­ecules 1 and 2, respectively. In each mol­ecule there is intra­molecular C—H⋯O hydrogen bond forming an *S*(6) ring motif. In mol­ecule 1 there is an intra­molecular N—H⋯π(pyrazole) inter­action and an intra­molecular C—H⋯π(pyrazole) inter­action present. Mol­ecule 1 is linked to mol­ecule 2 by a C—H⋯π(benzene ring) inter­action. An intra­molecular N—H⋯N hydrogen bond and an intra­molecular C—H⋯N hydrogen bond are also present in mol­ecule 2. In the crystal, the three components are linked by O_water_—H⋯N, N—H⋯O_water_ and N—H⋯N hydrogen bonds, forming chains along the [100] direction. The chains are linked by C—H⋯O and C—H⋯N hydrogen bonds, forming layers parallel to the *ab* plane. Finally, the layers are linked by C—H⋯π inter­actions, forming a three-dimensional structure.

## Abstract 2

The asymmetric unit of the title compound contains two independent organic mol­ecules which differ primarily in the dihedral angle between the aromatic rings, *viz*. 7.79 (7) and 29.89 (7)°. In the crystal, the components are linked by O_water_—H⋯N, N—H⋯O_water_ and N—H⋯N hydrogen bonds, forming chains along the [100] direction. The chains are linked by C—H⋯O and C—H⋯N hydrogen bonds, forming layers parallel to the *ab* plane. Finally, the layers are linked by C—H⋯π inter­actions, forming a three-dimensional structure.

## Chemical context   

Pyrazole derivatives are biologically active heterocyclic compounds (Karrouchi *et al.*, 2018 ▸). This compound class has been the topic of numerous pharmaceutical studies with members being used for their medicinal properties such as anti-inflammatory (Abdellatif *et al.*, 2018 ▸), anti­diabetic (Pillai *et al.*, 2019 ▸), anti­viral (El-Sabbagh *et al.*, 2009 ▸), analgesic (Karrouchi *et al.*, 2016 ▸), anti­tumoral (Guillén *et al.*, 2017 ▸), catecholase (Karrouchi *et al.*, 2018 ▸), and even as insecticides (Shi *et al.*, 2017 ▸). In particular, pyrazolylacetamide derivatives are widely studied with increasing inter­est because of their anti­oxidant (Chkirate *et al.*, 2019 ▸), antagonist (Chambers *et al.*, 2010 ▸; Beswick *et al.*, 2010 ▸) and anti-inflammatory (Sunder *et al.*, 2013 ▸), as well as their anti­microbial potential and anti­cancer (Dev *et al.*, 2017 ▸) activities. The present study is a continuation of the synthesis of the methyl-pyrazolyl-acétamide derivatives performed by our team (Chkirate *et al.*, 2001 ▸, 2017*a* ▸,*b* ▸). In this work, we prepared the title compound, by reacting *N*-2-amino­phenyl-5-methyl-pyrazol-3-ylacetamide with 4-methyl­benzaldehyde in acetone. We report herein on its crystal and mol­ecular structures along with the Hirshfeld surface analysis.
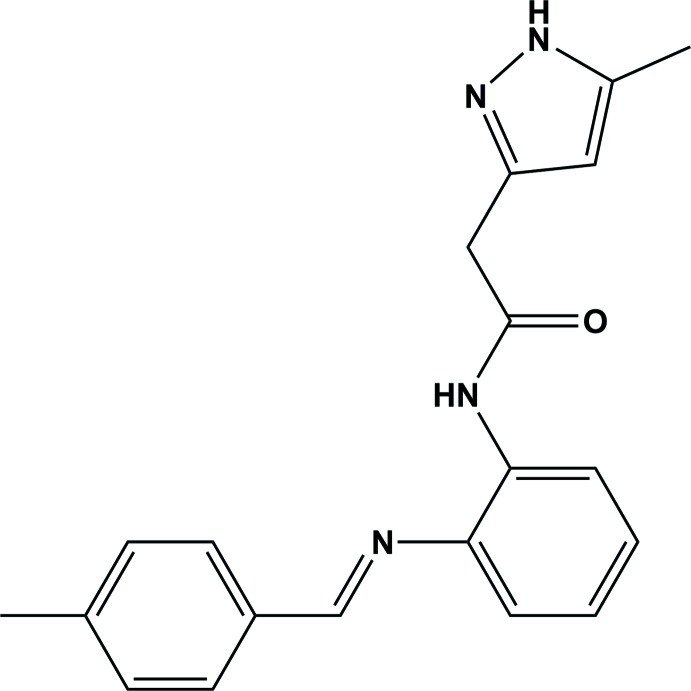


## Structural commentary   

**Figure 1:**
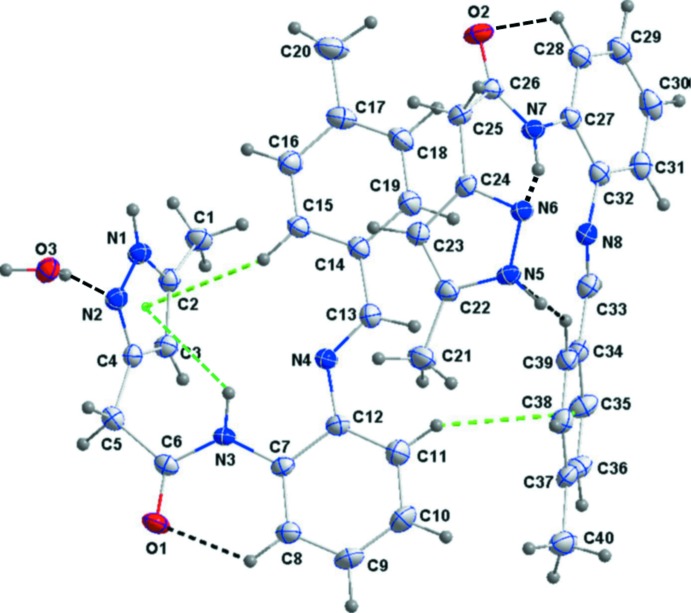
The asymmetric unit of the title compound, with the labelling scheme and 50% probability ellipsoids. The C—H⋯O and C—H⋯N hydrogen bonds are shown as black dashed lines and the C—H⋯π(ring) inter­actions by green dashed lines (see Table 1 ▸ for details).

**Figure 2:**
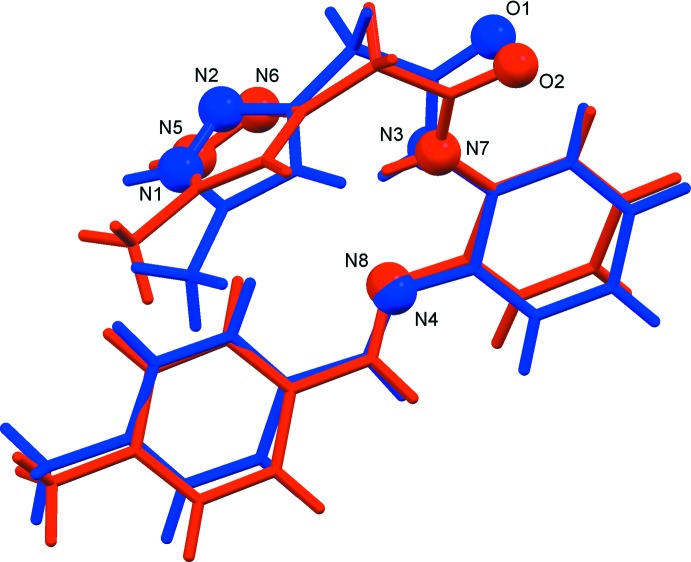
A mol­ecular overlap view of inverted mol­ecule 2 (red) on mol­ecule 1 (blue).

The mol­ecular structure of the title compound is illustrated in Fig. 1 ▸. The asymmetric unit contains two independent organic mol­ecules (1 and 2) and a water mol­ecule. The organic mol­ecules differ primarily in the dihedral angles between the aromatic rings. In the mol­ecule 1, the C7–C12 benzene ring is inclined to the C14–C19 benzene ring by 7.79 (7)°, while the corresponding angle in mol­ecule 2 is 29.89 (7)°. The mol­ecule overlay in Fig. 2 ▸, a view of inverted mol­ecule 2 on mol­ecule 1, illustrates the difference in the conformations of the two mol­ecules, with an r.m.s. deviation of 0.58 Å for the 25 non-hydrogen atoms.

The pyrazole ring (N1/N2/C2–C4) in mol­ecule 1 is inclined to the benzene rings (C7–C12 and C14–C19) by 70.83 (8) and 76.79 (8)°, respectively. The corresponding dihedral angles in mol­ecule 2, involving the N5/N6/C22–C24 pyrazole ring and the C27–C32 and C34–C39 benzene rings, are 68.47 (8) and 81.91 (8)°, respectively. In both mol­ecules there is an intra­molecular C—H⋯O hydrogen bond forming an *S*(6) ring motif (Fig. 1 ▸, Table 1 ▸). In the pyrazole rings, the N1—N2 and N5—N6 bond lengths are essentially equivalent, *viz*. 1.3595 (16) and 1.3596 (16) Å, respectively.

In mol­ecule 1, an intra­molecular N—H⋯π(pyrazole) inter­action and an intra­molecular C—H⋯π(pyrazole) inter­action are present (Fig. 1 ▸, Table 1 ▸). Mol­ecule 1 is linked to mol­ecule 2 by a C—H⋯π(benzene ring) inter­action, and in mol­ecule 2 an N—H⋯N and a C—H⋯N hydrogen bond are present (Fig. 1 ▸, Table 1 ▸).

## Supra­molecular features   

**Figure 3:**
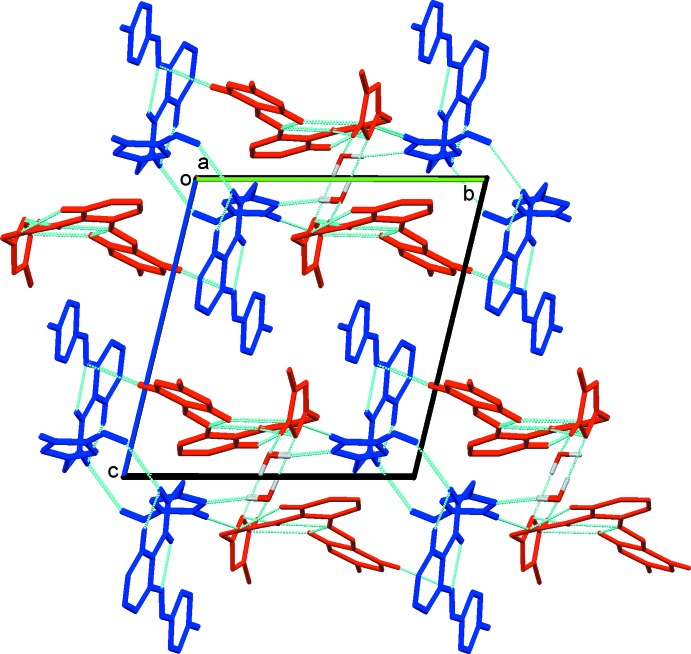
A partial view along the *a* axis of the crystal packing of the title compound. The hydrogen bonds are shown as dashed lines (see Table 1 ▸; colour code: mol­ecule 1 is blue, mol­ecule 2 is red).

**Figure 4:**
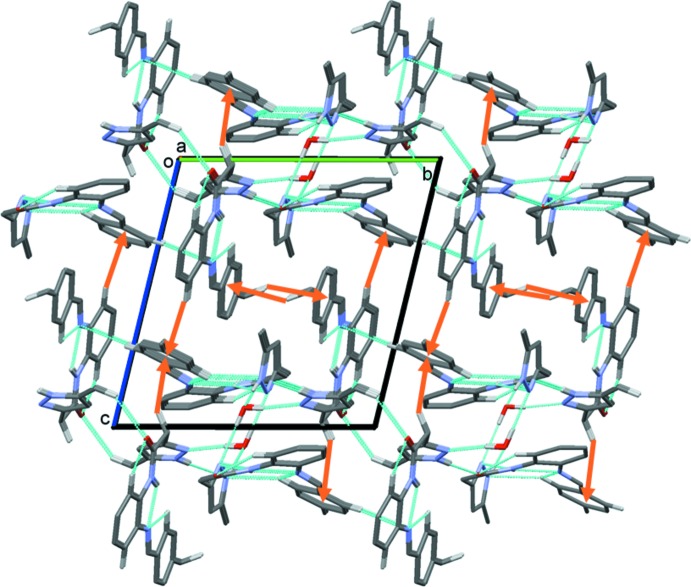
A view along the *a* axis of the crystal packing of the title compound. The hydrogen bonds are shown as dashed lines and the C—H⋯π inter­actions as orange arrows (see Table 1 ▸).

In the crystal, the three components are linked by O_water_—H⋯N and N—H⋯O_water_ hydrogen bonds, and by N—H⋯N hydrogen bonds, forming chains propagating along the *a*-axis direction; see Fig. 3 ▸. Full details of the various intra- and inter­molecular inter­actions are given in Table 1 ▸. The chains are linked by C—H⋯O and C—H⋯N hydrogen bonds, forming layers parallel to the *ab* plane (Fig. 3 ▸). Finally the layers are linked by C—H⋯π inter­actions, forming a three-dimensional structure (Fig. 4 ▸).

## Database survey   

A search of the Cambridge Structural Database (CSD, version 5.39, update May 2018; Groom *et al.*, 2016 ▸), for *N*-[2-(methyl­ene­amino)­phen­yl]acetamides gave many hits. A search for the substructure [2-(benzyl­idene­amino)­phen­yl]acetamide gave 19 hits, some of which are metal complexes. The structures most similar to the title compound include: *N*-(2-{[(2-hy­droxy­phen­yl)methyl­idene]amino}­phen­yl)-2,2-di­methyl­propanamide (POSPET; Kämpfe *et al.*, 2009 ▸), *o*-benzamido-*N*-(*o*-nitro­benzilidine)aniline (RIHHPF; Aldo­shin *et al.*, 1995 ▸), *o*-(*p*-nitro­benzamido)-*N*-(*o*-nitro­benzil­idene)aniline (RIHHUL; Aldoshin *et al.*, 1995 ▸), and *o*-(adamantanecarbamido)-*N*-(*m*-nitro­benzil­idene)aniline (RIHJAT; Aldoshin *et al.*, 1995 ▸). There is an extremely large difference in the dihedral angles between the two aryl rings in these compounds, *viz.* 44.36 (5)° for POSPET, 16.2 (2)° for RIHHOF, 41.81 (14)° for RIHHUL and 11.2 (4)° in RIHJAT. The dihedral angles between the aromatic rings in the title compound are 7.79 (7) and 29.89 (7)° in mol­ecules 1 and 2, respectively.

A search for {2-[(1-phenyl­ethyl­idene)amino]­phen­yl}acet­amides gave an inter­esting hit, namely that for *N*-(2-{[(1*E*)-1-(2-hy­droxy­phen­yl)ethyl­idene]amino}­phen­yl)-2-meth­oxy­acet­amide (TIGQIK; Yildirim *et al.*, 2007 ▸). Here the two aryl rings are almost coplanar with a dihedral angle of 1.2 (4)°. This small angle can be explained by the presence of an intra­molecular N—H⋯N hydrogen bond, rather than a weak C—H⋯O hydrogen bond as is present in the two mol­ecules of the title compound.

## Hirshfeld surface analysis   

**Figure 5:**
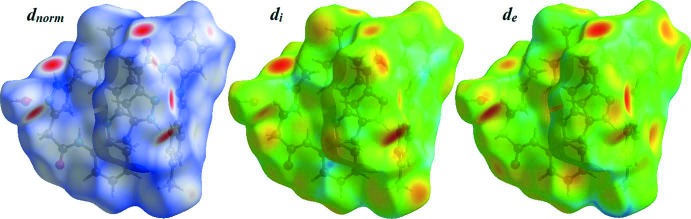
The Hirshfeld surface of the title compound mapped over *d*_norm_, *d*_i_ and *d*_e_.

**Figure 6:**
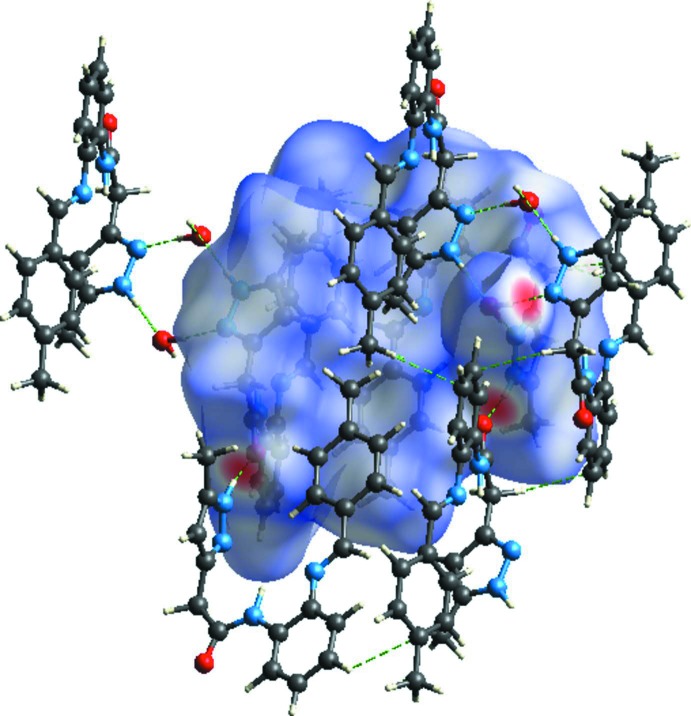
Hirshfeld surfaces mapped over *d*_norm_ to visualize the inter­molecular O—H⋯N and N—H⋯O hydrogen bonds and C—H⋯π inter­actions in the title compound.

The Hirshfeld surface analyse was carried out using *CrystalExplorer17.5* (Turner *et al.*, 2017 ▸). The Hirshfeld surfaces and their associated two-dimensional fingerprint plots were used to qu­antify the various inter­molecular inter­actions in the title compound. A 2D fingerprint graph gives a summary of the inter­molecular contacts in the crystal. The Hirshfeld surfaces mapped over *d*_norm_, *d*_e_ and *d*_i_ are illustrated in Fig. 5 ▸. The mol­ecular Hirshfeld surfaces were generated using a standard (high) surface resolution with the three-dimensional *d*_norm_ surfaces mapped over a fixed colour scale of −0.635 (red) to 1.583 (blue) Å. Fig. 6 ▸ illustrates the inter­molecular O—H⋯N, N—H⋯O and C—H⋯π inter­actions (Table 1 ▸) of the title compound with *d*_norm_ mapped on the Hirshfeld surface.

**Figure 7:**
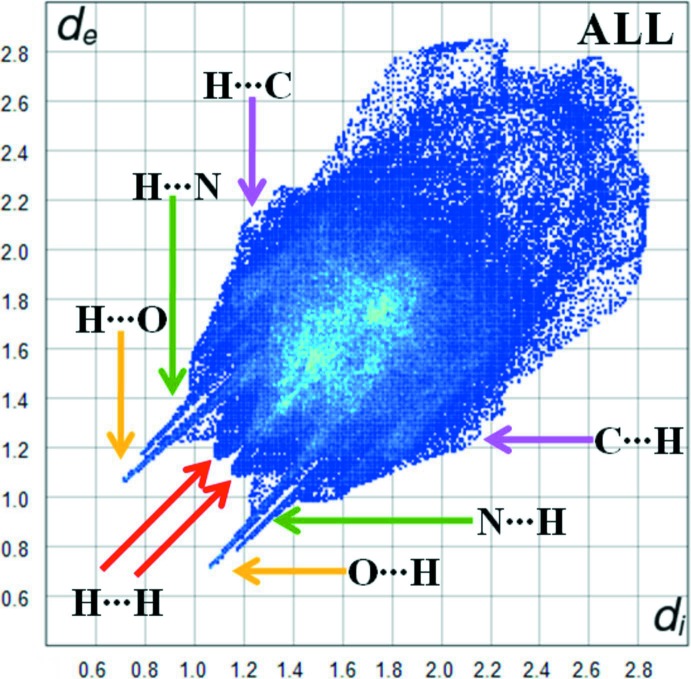
Two-dimensional fingerprint plot for the sum of the contacts contributing to the Hirshfeld surface.

**Figure 8:**
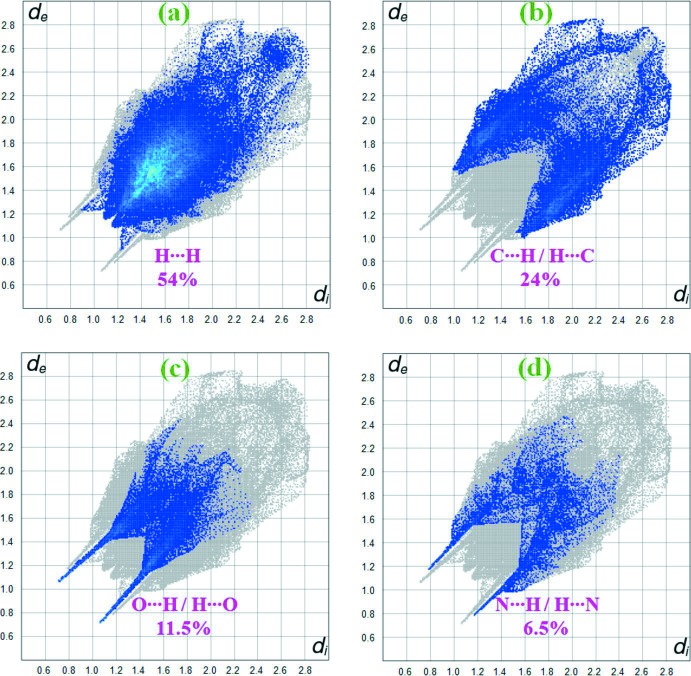
Two-dimensional fingerprint plots for the (*a*) H⋯H (54%), (*b*) C⋯H/H⋯C (24%), (*c*) O⋯H/H⋯O (11.5%) and (*d*) N⋯H/H⋯N (6.5%) contacts in the title compound.

Fig. 7 ▸ shows the two-dimensional fingerprint plot of the sum of the contacts contributing to the Hirshfeld surface represented in normal mode. Fig. 8 ▸ *a* (H⋯H) illustrates the two-dimensional fingerprint of the (*d_i_*, *d_e_*) points associated with hydrogen atoms. It is characterized by an end point that points to the origin and corresponds to *d_i_* = *d_e_* = 1.08 Å, which indicates the presence of the H⋯H contacts in this study (54%). Fig. 8 ▸ *b* (C⋯H/H⋯C) shows the contacts between the carbon atoms inside the surface and the hydrogen atoms outside the surface of Hirshfeld and *vice versa* (24%). The O⋯H/H⋯O (11.5%) plot shows two symmetrical wings on the left and right sides (Fig. 8 ▸ *c*). The N⋯H/H⋯N inter­actions (6.5%) are visualized in Fig. 8 ▸ *d*.

## Synthesis and crystallization   

The title compound was prepared by stirring *N*-2-amino­phenyl-5-methyl­pyrazol-3-ylacetamide (0.5 g, 2.2 mmol) with 4-methyl­benzaldehyde (1.05 g, 8.8 mmol) in acetone (50 ml) for 3 h. The solvent was evaporated under vacuum, and then water was added. The precipitate formed was filtered under vacuum and purified through silica gel column chromatography using hexa­ne/ethyl acetate (6/4, *v*/*v*), yielding colourless rod-like crystals of the title compound (yield 63%).

## Refinement   

Crystal data, data collection and structure refinement details are summarized in Table 2 ▸. All the H atoms were located in difference-Fourier maps and freely refined.

## Supplementary Material

Crystal structure: contains datablock(s) global, I. DOI: 10.1107/S2056989018017747/xu5954sup1.cif

Structure factors: contains datablock(s) I. DOI: 10.1107/S2056989018017747/xu5954Isup2.hkl

Supporting information file. DOI: 10.1107/S2056989018017747/xu5954Isup3.cml

CCDC reference: 1885214

Additional supporting information:  crystallographic information; 3D view; checkCIF report

## Figures and Tables

**Table 1:** Hydrogen-bond geometry (Å, °) *Cg*1, *Cg*3 and *Cg*6 are the centroids of the N1/N2/C2–C4, C14–C19 and C34–C39 rings, respectively.

*D*—H⋯*A*	*D*—H	H⋯*A*	*D*⋯*A*	*D*—H⋯*A*
N1—H1⋯O2^i^	0.92 (2)	1.88 (2)	2.7863 (18)	169 (2)
O3—H3*B*⋯N2	0.92 (2)	1.91 (2)	2.8047 (19)	165 (2)
O3—H3*C*⋯N6^ii^	0.87 (2)	2.09 (2)	2.9530 (18)	174 (2)
N5—H5⋯O3^iii^	0.93 (2)	1.878 (19)	2.8014 (18)	173 (2)
N7—H7⋯N6	0.91 (2)	2.447 (17)	3.1314 (19)	132.6 (13)
C1—H1*A*⋯O1^iv^	0.99 (2)	2.56 (2)	3.436 (2)	146.9 (15)
C8—H8⋯O1	0.98 (2)	2.228 (15)	2.858 (2)	120.8 (12)
C28—H28⋯O2	1.01 (2)	2.265 (18)	2.890 (2)	118.5 (13)
C35—H35⋯N4^v^	0.99 (2)	2.532 (18)	3.451 (2)	155.2 (13)
N3—H3*A*⋯*Cg*1	0.91 (2)	2.999 (15)	3.6216 (17)	127.4 (12)
C5—H5*B*⋯*Cg*6^ii^	1.00 (2)	2.820 (16)	3.7171 (18)	149.0 (12)
C11—H11⋯*Cg*6	0.98 (2)	2.837 (19)	3.713 (2)	149.8 (14)
C15—H15⋯*Cg*1	0.99 (2)	2.913 (15)	3.7979 (19)	149.8 (12)
C20—H20*B*⋯*Cg*3^i^	1.00 (2)	2.88 (2)	3.772 (3)	148.9 (16)

Symmetry codes: (i) 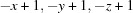; (ii) 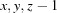; (iii) 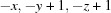; (iv) 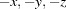; (v) 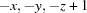.

**Table 2:** Experimental details

Crystal data	
Chemical formula	C_20_H_20_N_4_O·0.5H_2_O
*M*_r_	341.41
Crystal system, space group	Triclinic, *P* 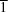
Temperature (K)	100
*a*, *b*, *c* (Å)	11.546 (3), 12.564 (3), 13.172 (3)
α, β, γ (°)	101.991 (3), 97.535 (3), 99.847 (3)
*V* (Å^3^)	1813.8 (7)
*Z*	4
Radiation type	Mo *K*α
μ (mm^−1^)	0.08
Crystal size (mm)	0.21 × 0.13 × 0.12

Data collection	
Diffractometer	Bruker SMART APEX CCD
Absorption correction	Multi-scan (*SADABS*; Krause *et al.*, 2015 ▸)
*T*_min_, *T*_max_	0.88, 0.99
No. of measured, independent and observed [*I* > 2σ(*I*)] reflections	17171, 8663, 5888
*R*_int_	0.033
(sin θ/λ)_max_ (Å^−1^)	0.664

Refinement	
*R*[*F* ^2^ > 2σ(*F* ^2^)], *wR*(*F* ^2^), *S*	0.046, 0.122, 1.04
No. of reflections	8663
No. of parameters	628
H-atom treatment	All H-atom parameters refined
Δρ_max_, Δρ_min_ (e Å^−3^)	0.28, −0.19

Computer programs: *APEX3* and *SAINT* (Bruker, 2016 ▸), *SHELXT* (Sheldrick, 2015*a* ▸), *SHELXL2018/1* (Sheldrick, 2015*b* ▸), *DIAMOND* (Brandenburg & Putz, 2012 ▸), *Mercury* (Macrae *et al.*, 2008 ▸), *PLATON* (Spek, 2009 ▸) and *publCIF* (Westrip, 2010 ▸).

## supplementary crystallographic information

### Crystal data

**Table:**

C_20_H_20_N_4_O·0.5H_2_O	*Z* = 4
*M**_r_* = 341.41	*F*(000) = 724
Triclinic, *P*1	*D*_x_ = 1.250 Mg m^−^^3^
*a* = 11.546 (3) Å	Mo *K*α radiation, λ = 0.71073 Å
*b* = 12.564 (3) Å	Cell parameters from 5130 reflections
*c* = 13.172 (3) Å	θ = 2.2–28.2°
α = 101.991 (3)°	µ = 0.08 mm^−^^1^
β = 97.535 (3)°	*T* = 100 K
γ = 99.847 (3)°	Rod, colourless
*V* = 1813.8 (7) Å^3^	0.21 × 0.13 × 0.12 mm

### Data collection

**Table:**

Bruker SMART APEX CCD diffractometer	8663 independent reflections
Radiation source: fine-focus sealed tube	5888 reflections with *I* > 2σ(*I*)
Graphite monochromator	*R*_int_ = 0.033
Detector resolution: 8.3333 pixels mm^-1^	θ_max_ = 28.2°, θ_min_ = 1.6°
ω scans	*h* = −15→15
Absorption correction: multi-scan (*SADABS*; Krause *et al.*, 2015)	*k* = −16→16
*T*_min_ = 0.88, *T*_max_ = 0.99	*l* = −17→17
17171 measured reflections	

### Refinement

**Table:**

Refinement on *F*^2^	Primary atom site location: structure-invariant direct methods
Least-squares matrix: full	Secondary atom site location: difference Fourier map
*R*[*F*^2^ > 2σ(*F*^2^)] = 0.046	Hydrogen site location: difference Fourier map
*wR*(*F*^2^) = 0.122	All H-atom parameters refined
*S* = 1.03	*w* = 1/[σ^2^(*F*_o_^2^) + (0.0564*P*)^2^] where *P* = (*F*_o_^2^ + 2*F*_c_^2^)/3
8663 reflections	(Δ/σ)_max_ < 0.001
628 parameters	Δρ_max_ = 0.28 e Å^−^^3^
0 restraints	Δρ_min_ = −0.19 e Å^−^^3^

### Special details

**Table:**

Experimental. The diffraction data were collected in three sets of 363 frames (0.5° width in ω) at φ = 0, 120 and 240°. A scan time of 60 sec/frame was used.
Geometry. All esds (except the esd in the dihedral angle between two l.s. planes) are estimated using the full covariance matrix. The cell esds are taken into account individually in the estimation of esds in distances, angles and torsion angles; correlations between esds in cell parameters are only used when they are defined by crystal symmetry. An approximate (isotropic) treatment of cell esds is used for estimating esds involving l.s. planes.
Refinement. Refinement of F^2^ against ALL reflections. The weighted R-factor wR and goodness of fit S are based on F^2^, conventional R-factors R are based on F, with F set to zero for negative F^2^. The threshold expression of F^2^ > 2sigma(F^2^) is used only for calculating R-factors(gt) etc. and is not relevant to the choice of reflections for refinement. R-factors based on F^2^ are statistically about twice as large as those based on F, and R- factors based on ALL data will be even larger.

### Fractional atomic coordinates and isotropic or equivalent isotropic displacement parameters (Å^2^)

**Table:**

	*x*	*y*	*z*	*U*_iso_*/*U*_eq_	
O1	−0.24038 (9)	0.13666 (9)	0.04235 (8)	0.0313 (3)	
N1	0.27104 (11)	0.26740 (10)	0.11862 (10)	0.0240 (3)	
H1	0.3426 (15)	0.3173 (14)	0.1367 (13)	0.040 (5)*	
N2	0.16821 (10)	0.29967 (10)	0.08749 (9)	0.0238 (3)	
N3	−0.09799 (11)	0.19107 (10)	0.19035 (9)	0.0213 (3)	
H3A	−0.0190 (13)	0.2168 (12)	0.2155 (12)	0.023 (4)*	
N4	0.02622 (10)	0.22466 (10)	0.38113 (9)	0.0233 (3)	
C1	0.35478 (14)	0.10461 (14)	0.14763 (14)	0.0284 (3)	
H1A	0.3326 (16)	0.0229 (16)	0.1186 (15)	0.051 (5)*	
H1B	0.3722 (16)	0.1144 (15)	0.2238 (16)	0.051 (5)*	
H1C	0.4241 (17)	0.1349 (15)	0.1255 (15)	0.052 (6)*	
C2	0.25432 (12)	0.15807 (11)	0.11593 (11)	0.0222 (3)	
C3	0.13432 (13)	0.11639 (12)	0.08140 (11)	0.0236 (3)	
H3	0.0949 (14)	0.0375 (13)	0.0687 (13)	0.036 (5)*	
C4	0.08456 (12)	0.20676 (11)	0.06429 (11)	0.0205 (3)	
C5	−0.04210 (13)	0.20825 (13)	0.02199 (11)	0.0229 (3)	
H5A	−0.0458 (12)	0.2852 (13)	0.0168 (12)	0.026 (4)*	
H5B	−0.0678 (13)	0.1591 (12)	−0.0510 (13)	0.027 (4)*	
C6	−0.13684 (12)	0.17440 (11)	0.08508 (11)	0.0213 (3)	
C7	−0.16340 (12)	0.16660 (11)	0.26803 (11)	0.0204 (3)	
C8	−0.28614 (13)	0.12593 (12)	0.24842 (13)	0.0257 (3)	
H8	−0.3322 (13)	0.1159 (12)	0.1777 (12)	0.026 (4)*	
C9	−0.34209 (15)	0.09808 (15)	0.32871 (14)	0.0366 (4)	
H9	−0.4304 (15)	0.0704 (13)	0.3126 (13)	0.038 (5)*	
C10	−0.27788 (15)	0.11076 (17)	0.42750 (15)	0.0452 (5)	
H10	−0.3172 (17)	0.0920 (15)	0.4852 (16)	0.055 (6)*	
C11	−0.15655 (15)	0.15421 (15)	0.44915 (14)	0.0375 (4)	
H11	−0.1124 (15)	0.1630 (14)	0.5198 (15)	0.047 (5)*	
C12	−0.09769 (12)	0.18343 (11)	0.37043 (11)	0.0226 (3)	
C13	0.09606 (13)	0.24843 (13)	0.46912 (12)	0.0265 (3)	
H13	0.0668 (15)	0.2413 (14)	0.5348 (14)	0.047 (5)*	
C14	0.22509 (13)	0.28361 (12)	0.47815 (11)	0.0246 (3)	
C15	0.27780 (13)	0.29677 (12)	0.39088 (12)	0.0262 (3)	
H15	0.2243 (13)	0.2821 (12)	0.3225 (12)	0.027 (4)*	
C16	0.39990 (14)	0.32762 (14)	0.40179 (13)	0.0312 (4)	
H16	0.4323 (14)	0.3411 (13)	0.3410 (13)	0.035 (5)*	
C17	0.47409 (13)	0.34649 (13)	0.49918 (12)	0.0297 (4)	
C18	0.42097 (15)	0.33447 (15)	0.58584 (13)	0.0354 (4)	
H18	0.4721 (15)	0.3483 (13)	0.6554 (14)	0.040 (5)*	
C19	0.29838 (14)	0.30374 (15)	0.57594 (13)	0.0350 (4)	
H19	0.2595 (15)	0.2958 (14)	0.6390 (14)	0.048 (5)*	
C20	0.60729 (15)	0.37970 (19)	0.50972 (17)	0.0432 (5)	
H20A	0.642 (2)	0.319 (2)	0.469 (2)	0.101 (9)*	
H20B	0.6300 (18)	0.4481 (18)	0.4833 (17)	0.072 (7)*	
H20C	0.647 (2)	0.3942 (19)	0.583 (2)	0.087 (8)*	
O2	0.52850 (9)	0.56026 (9)	0.82428 (9)	0.0319 (3)	
N5	0.03382 (10)	0.52348 (10)	0.81415 (10)	0.0228 (3)	
H5	−0.0253 (16)	0.5056 (14)	0.8528 (14)	0.045 (5)*	
N6	0.15011 (10)	0.55805 (9)	0.86144 (9)	0.0210 (3)	
N7	0.36667 (11)	0.43953 (10)	0.84380 (9)	0.0218 (3)	
H7	0.2865 (15)	0.4290 (13)	0.8401 (13)	0.037 (5)*	
N8	0.21561 (10)	0.25089 (10)	0.83158 (9)	0.0234 (3)	
C21	−0.10166 (15)	0.48113 (16)	0.64252 (16)	0.0361 (4)	
H21A	−0.163 (2)	0.4882 (19)	0.6879 (19)	0.086 (8)*	
H21B	−0.1168 (17)	0.4019 (18)	0.6063 (16)	0.065 (6)*	
H21C	−0.1135 (15)	0.5315 (15)	0.5943 (15)	0.052 (5)*	
C22	0.01855 (13)	0.51647 (11)	0.70978 (12)	0.0245 (3)	
C23	0.13064 (13)	0.54799 (12)	0.68677 (12)	0.0255 (3)	
H23	0.1522 (13)	0.5523 (12)	0.6183 (13)	0.030 (4)*	
C24	0.20896 (12)	0.57328 (11)	0.78264 (11)	0.0209 (3)	
C25	0.34089 (13)	0.61544 (12)	0.80340 (13)	0.0236 (3)	
H25A	0.3667 (13)	0.6811 (12)	0.8659 (12)	0.027 (4)*	
H25B	0.3656 (13)	0.6432 (12)	0.7431 (13)	0.031 (4)*	
C26	0.42068 (12)	0.53575 (12)	0.82503 (11)	0.0228 (3)	
C27	0.41856 (12)	0.35211 (11)	0.86889 (11)	0.0213 (3)	
C28	0.54056 (13)	0.36037 (13)	0.89766 (12)	0.0270 (3)	
H28	0.5982 (15)	0.4314 (15)	0.8988 (13)	0.044 (5)*	
C29	0.58176 (14)	0.27065 (13)	0.92452 (12)	0.0295 (4)	
H29	0.6701 (15)	0.2783 (13)	0.9482 (13)	0.040 (5)*	
C30	0.50266 (14)	0.17427 (14)	0.92401 (12)	0.0301 (4)	
H30	0.5291 (14)	0.1144 (13)	0.9446 (13)	0.032 (4)*	
C31	0.38173 (14)	0.16498 (13)	0.89364 (12)	0.0272 (3)	
H31	0.3233 (13)	0.0982 (12)	0.8945 (11)	0.022 (4)*	
C32	0.33787 (12)	0.25261 (12)	0.86328 (11)	0.0229 (3)	
C33	0.14467 (13)	0.15882 (12)	0.78537 (12)	0.0245 (3)	
H33	0.1751 (14)	0.0909 (13)	0.7694 (12)	0.033 (4)*	
C34	0.01636 (13)	0.14971 (12)	0.75516 (11)	0.0234 (3)	
C35	−0.05642 (14)	0.04580 (13)	0.70870 (13)	0.0303 (4)	
H35	−0.0215 (14)	−0.0207 (14)	0.6920 (13)	0.037 (5)*	
C36	−0.17876 (14)	0.03414 (13)	0.68350 (13)	0.0324 (4)	
H36	−0.2277 (15)	−0.0379 (14)	0.6534 (13)	0.040 (5)*	
C37	−0.23271 (13)	0.12516 (12)	0.70395 (12)	0.0269 (3)	
C38	−0.15965 (14)	0.22914 (13)	0.74951 (12)	0.0263 (3)	
H38	−0.1943 (13)	0.2927 (12)	0.7645 (12)	0.026 (4)*	
C39	−0.03736 (13)	0.24180 (12)	0.77441 (12)	0.0246 (3)	
H39	0.0132 (12)	0.3148 (12)	0.8089 (11)	0.020 (4)*	
C40	−0.36608 (15)	0.11183 (16)	0.67982 (15)	0.0358 (4)	
H40A	−0.4014 (16)	0.1124 (14)	0.7448 (15)	0.049 (5)*	
H40B	−0.3951 (17)	0.1743 (17)	0.6509 (15)	0.060 (6)*	
H40C	−0.4044 (15)	0.0410 (15)	0.6284 (14)	0.045 (5)*	
O3	0.15336 (9)	0.51707 (9)	0.07448 (9)	0.0266 (2)	
H3B	0.1689 (17)	0.4475 (17)	0.0720 (16)	0.060 (6)*	
H3C	0.1580 (19)	0.5294 (18)	0.0125 (19)	0.075 (7)*	

### Atomic displacement parameters (Å^2^)

**Table:**

	*U*^11^	*U*^22^	*U*^33^	*U*^12^	*U*^13^	*U*^23^
O1	0.0194 (6)	0.0403 (6)	0.0291 (6)	0.0035 (5)	−0.0040 (4)	0.0042 (5)
N1	0.0183 (6)	0.0269 (7)	0.0278 (7)	0.0042 (5)	0.0049 (5)	0.0087 (5)
N2	0.0210 (6)	0.0262 (7)	0.0258 (7)	0.0054 (5)	0.0061 (5)	0.0083 (5)
N3	0.0147 (6)	0.0251 (6)	0.0221 (6)	0.0014 (5)	0.0006 (5)	0.0051 (5)
N4	0.0198 (6)	0.0263 (6)	0.0218 (6)	0.0023 (5)	0.0017 (5)	0.0046 (5)
C1	0.0214 (8)	0.0325 (9)	0.0330 (9)	0.0088 (7)	0.0041 (7)	0.0089 (7)
C2	0.0226 (8)	0.0239 (7)	0.0215 (7)	0.0060 (6)	0.0061 (6)	0.0059 (6)
C3	0.0229 (8)	0.0215 (8)	0.0261 (8)	0.0042 (6)	0.0028 (6)	0.0060 (6)
C4	0.0216 (7)	0.0229 (7)	0.0173 (7)	0.0041 (6)	0.0047 (6)	0.0047 (6)
C5	0.0245 (8)	0.0233 (8)	0.0202 (8)	0.0061 (6)	0.0011 (6)	0.0045 (6)
C6	0.0195 (7)	0.0200 (7)	0.0239 (7)	0.0073 (6)	0.0010 (6)	0.0035 (6)
C7	0.0191 (7)	0.0173 (7)	0.0249 (7)	0.0027 (5)	0.0047 (6)	0.0052 (6)
C8	0.0202 (8)	0.0253 (8)	0.0304 (9)	0.0028 (6)	0.0017 (6)	0.0068 (6)
C9	0.0198 (8)	0.0471 (10)	0.0421 (10)	−0.0010 (7)	0.0073 (7)	0.0142 (8)
C10	0.0302 (10)	0.0678 (13)	0.0387 (11)	−0.0023 (9)	0.0134 (8)	0.0201 (9)
C11	0.0300 (9)	0.0530 (11)	0.0283 (9)	0.0009 (8)	0.0054 (7)	0.0127 (8)
C12	0.0185 (7)	0.0235 (7)	0.0252 (8)	0.0030 (6)	0.0046 (6)	0.0049 (6)
C13	0.0256 (8)	0.0314 (8)	0.0210 (8)	0.0019 (6)	0.0036 (6)	0.0062 (6)
C14	0.0236 (8)	0.0261 (8)	0.0214 (7)	0.0027 (6)	0.0013 (6)	0.0032 (6)
C15	0.0219 (8)	0.0316 (8)	0.0209 (8)	0.0015 (6)	0.0003 (6)	0.0026 (6)
C16	0.0265 (9)	0.0382 (9)	0.0261 (8)	0.0015 (7)	0.0061 (7)	0.0045 (7)
C17	0.0211 (8)	0.0305 (8)	0.0325 (9)	0.0031 (6)	0.0000 (6)	0.0009 (7)
C18	0.0273 (9)	0.0488 (10)	0.0250 (9)	0.0033 (8)	−0.0044 (7)	0.0069 (7)
C19	0.0281 (9)	0.0509 (11)	0.0227 (8)	0.0005 (8)	0.0020 (7)	0.0086 (7)
C20	0.0216 (9)	0.0559 (13)	0.0444 (12)	0.0013 (8)	−0.0009 (8)	0.0038 (10)
O2	0.0173 (5)	0.0331 (6)	0.0444 (7)	0.0004 (4)	0.0059 (5)	0.0104 (5)
N5	0.0167 (6)	0.0225 (6)	0.0303 (7)	0.0045 (5)	0.0051 (5)	0.0081 (5)
N6	0.0179 (6)	0.0208 (6)	0.0243 (6)	0.0033 (5)	0.0036 (5)	0.0058 (5)
N7	0.0159 (6)	0.0232 (6)	0.0264 (7)	0.0031 (5)	0.0035 (5)	0.0067 (5)
N8	0.0232 (6)	0.0250 (6)	0.0223 (6)	0.0029 (5)	0.0049 (5)	0.0070 (5)
C21	0.0273 (9)	0.0372 (10)	0.0410 (10)	0.0015 (8)	−0.0063 (8)	0.0148 (8)
C22	0.0232 (8)	0.0210 (7)	0.0274 (8)	0.0022 (6)	−0.0007 (6)	0.0067 (6)
C23	0.0265 (8)	0.0258 (8)	0.0238 (8)	0.0033 (6)	0.0041 (6)	0.0069 (6)
C24	0.0220 (7)	0.0171 (7)	0.0251 (8)	0.0047 (6)	0.0058 (6)	0.0067 (6)
C25	0.0199 (8)	0.0230 (8)	0.0271 (8)	0.0002 (6)	0.0055 (6)	0.0067 (6)
C26	0.0190 (7)	0.0250 (7)	0.0210 (7)	0.0006 (6)	0.0025 (6)	0.0018 (6)
C27	0.0218 (7)	0.0254 (7)	0.0169 (7)	0.0061 (6)	0.0032 (6)	0.0044 (6)
C28	0.0230 (8)	0.0341 (9)	0.0231 (8)	0.0066 (7)	0.0031 (6)	0.0049 (6)
C29	0.0256 (8)	0.0413 (9)	0.0231 (8)	0.0133 (7)	0.0035 (7)	0.0061 (7)
C30	0.0357 (9)	0.0352 (9)	0.0244 (8)	0.0190 (8)	0.0059 (7)	0.0081 (7)
C31	0.0335 (9)	0.0268 (8)	0.0225 (8)	0.0081 (7)	0.0066 (7)	0.0056 (6)
C32	0.0234 (8)	0.0265 (8)	0.0194 (7)	0.0078 (6)	0.0052 (6)	0.0035 (6)
C33	0.0271 (8)	0.0221 (8)	0.0262 (8)	0.0056 (6)	0.0081 (6)	0.0071 (6)
C34	0.0264 (8)	0.0217 (7)	0.0228 (8)	0.0023 (6)	0.0074 (6)	0.0071 (6)
C35	0.0287 (9)	0.0219 (8)	0.0394 (9)	0.0023 (7)	0.0104 (7)	0.0049 (7)
C36	0.0302 (9)	0.0244 (8)	0.0378 (10)	−0.0046 (7)	0.0090 (7)	0.0033 (7)
C37	0.0246 (8)	0.0305 (8)	0.0257 (8)	−0.0014 (6)	0.0084 (6)	0.0101 (6)
C38	0.0286 (8)	0.0245 (8)	0.0275 (8)	0.0054 (7)	0.0061 (6)	0.0094 (6)
C39	0.0274 (8)	0.0211 (7)	0.0242 (8)	0.0011 (6)	0.0033 (6)	0.0068 (6)
C40	0.0256 (9)	0.0435 (11)	0.0355 (10)	0.0003 (8)	0.0096 (7)	0.0061 (8)
O3	0.0263 (6)	0.0266 (6)	0.0310 (6)	0.0078 (5)	0.0085 (5)	0.0116 (5)

### Geometric parameters (Å, º)

**Table:**

O1—C6	1.2205 (16)	N5—C22	1.3453 (19)
N1—C2	1.3464 (18)	N5—N6	1.3596 (16)
N1—N2	1.3595 (16)	N5—H5	0.928 (18)
N1—H1	0.918 (17)	N6—C24	1.3410 (17)
N2—C4	1.3333 (17)	N7—C26	1.3484 (18)
N3—C6	1.3608 (18)	N7—C27	1.4137 (18)
N3—C7	1.4021 (18)	N7—H7	0.906 (17)
N3—H3A	0.907 (15)	N8—C33	1.2775 (18)
N4—C13	1.2695 (19)	N8—C32	1.4137 (18)
N4—C12	1.4136 (18)	C21—C22	1.487 (2)
C1—C2	1.492 (2)	C21—H21A	0.99 (3)
C1—H1A	0.993 (19)	C21—H21B	0.99 (2)
C1—H1B	0.974 (19)	C21—H21C	1.00 (2)
C1—H1C	0.935 (19)	C22—C23	1.379 (2)
C2—C3	1.374 (2)	C23—C24	1.396 (2)
C3—C4	1.4019 (19)	C23—H23	0.976 (16)
C3—H3	0.987 (16)	C24—C25	1.493 (2)
C4—C5	1.4999 (19)	C25—C26	1.516 (2)
C5—C6	1.515 (2)	C25—H25A	1.008 (16)
C5—H5A	0.991 (15)	C25—H25B	0.987 (16)
C5—H5B	1.004 (16)	C27—C28	1.390 (2)
C7—C8	1.3920 (19)	C27—C32	1.408 (2)
C7—C12	1.4134 (19)	C28—C29	1.388 (2)
C8—C9	1.383 (2)	C28—H28	1.014 (18)
C8—H8	0.982 (15)	C29—C30	1.385 (2)
C9—C10	1.374 (2)	C29—H29	1.009 (17)
C9—H9	0.998 (16)	C30—C31	1.379 (2)
C10—C11	1.382 (2)	C30—H30	0.935 (16)
C10—H10	0.98 (2)	C31—C32	1.396 (2)
C11—C12	1.389 (2)	C31—H31	0.986 (14)
C11—H11	0.976 (18)	C33—C34	1.461 (2)
C13—C14	1.461 (2)	C33—H33	0.971 (16)
C13—H13	0.984 (18)	C34—C35	1.394 (2)
C14—C19	1.395 (2)	C34—C39	1.397 (2)
C14—C15	1.395 (2)	C35—C36	1.383 (2)
C15—C16	1.377 (2)	C35—H35	0.985 (16)
C15—H15	0.986 (15)	C36—C37	1.388 (2)
C16—C17	1.397 (2)	C36—H36	0.955 (17)
C16—H16	0.960 (17)	C37—C38	1.394 (2)
C17—C18	1.388 (2)	C37—C40	1.504 (2)
C17—C20	1.503 (2)	C38—C39	1.381 (2)
C18—C19	1.383 (2)	C38—H38	0.949 (15)
C18—H18	0.987 (17)	C39—H39	0.981 (14)
C19—H19	1.009 (18)	C40—H40A	0.994 (19)
C20—H20A	1.02 (3)	C40—H40B	1.03 (2)
C20—H20B	1.00 (2)	C40—H40C	0.992 (18)
C20—H20C	0.98 (3)	O3—H3B	0.92 (2)
O2—C26	1.2322 (17)	O3—H3C	0.87 (2)
			
C2—N1—N2	112.77 (12)	C22—N5—H5	126.5 (11)
C2—N1—H1	126.3 (10)	N6—N5—H5	120.8 (11)
N2—N1—H1	120.9 (10)	C24—N6—N5	104.31 (11)
C4—N2—N1	104.56 (11)	C26—N7—C27	128.61 (12)
C6—N3—C7	128.49 (12)	C26—N7—H7	116.3 (10)
C6—N3—H3A	118.4 (9)	C27—N7—H7	115.1 (10)
C7—N3—H3A	113.0 (9)	C33—N8—C32	119.62 (13)
C13—N4—C12	122.39 (13)	C22—C21—H21A	109.1 (13)
C2—C1—H1A	110.3 (10)	C22—C21—H21B	110.9 (11)
C2—C1—H1B	111.5 (11)	H21A—C21—H21B	105.8 (18)
H1A—C1—H1B	105.2 (15)	C22—C21—H21C	111.2 (10)
C2—C1—H1C	110.8 (11)	H21A—C21—H21C	105.3 (16)
H1A—C1—H1C	110.7 (15)	H21B—C21—H21C	114.0 (16)
H1B—C1—H1C	108.1 (15)	N5—C22—C23	106.25 (13)
N1—C2—C3	105.96 (12)	N5—C22—C21	121.78 (14)
N1—C2—C1	121.99 (13)	C23—C22—C21	131.97 (15)
C3—C2—C1	132.05 (14)	C22—C23—C24	105.66 (13)
C2—C3—C4	105.94 (13)	C22—C23—H23	128.0 (9)
C2—C3—H3	125.1 (9)	C24—C23—H23	126.3 (9)
C4—C3—H3	128.9 (9)	N6—C24—C23	111.07 (13)
N2—C4—C3	110.77 (12)	N6—C24—C25	120.87 (13)
N2—C4—C5	120.30 (12)	C23—C24—C25	128.03 (13)
C3—C4—C5	128.88 (13)	C24—C25—C26	118.03 (12)
C4—C5—C6	117.61 (12)	C24—C25—H25A	111.9 (8)
C4—C5—H5A	107.9 (8)	C26—C25—H25A	104.5 (8)
C6—C5—H5A	106.3 (8)	C24—C25—H25B	109.9 (9)
C4—C5—H5B	110.6 (8)	C26—C25—H25B	106.3 (9)
C6—C5—H5B	106.5 (8)	H25A—C25—H25B	105.3 (12)
H5A—C5—H5B	107.4 (12)	O2—C26—N7	123.63 (14)
O1—C6—N3	123.85 (13)	O2—C26—C25	120.04 (13)
O1—C6—C5	120.84 (13)	N7—C26—C25	116.33 (12)
N3—C6—C5	115.30 (12)	C28—C27—C32	120.48 (13)
C8—C7—N3	123.57 (13)	C28—C27—N7	123.96 (13)
C8—C7—C12	119.98 (13)	C32—C27—N7	115.56 (12)
N3—C7—C12	116.44 (12)	C29—C28—C27	119.24 (15)
C9—C8—C7	119.49 (15)	C29—C28—H28	120.9 (10)
C9—C8—H8	120.4 (9)	C27—C28—H28	119.9 (10)
C7—C8—H8	120.1 (9)	C30—C29—C28	120.68 (15)
C10—C9—C8	120.69 (15)	C30—C29—H29	120.4 (9)
C10—C9—H9	121.6 (10)	C28—C29—H29	118.8 (9)
C8—C9—H9	117.7 (10)	C31—C30—C29	120.25 (15)
C9—C10—C11	120.54 (16)	C31—C30—H30	118.3 (10)
C9—C10—H10	121.1 (11)	C29—C30—H30	121.5 (10)
C11—C10—H10	118.3 (11)	C30—C31—C32	120.37 (15)
C10—C11—C12	120.21 (16)	C30—C31—H31	121.9 (8)
C10—C11—H11	119.5 (10)	C32—C31—H31	117.7 (8)
C12—C11—H11	120.3 (11)	C31—C32—C27	118.86 (13)
C11—C12—C7	119.00 (13)	C31—C32—N8	124.36 (13)
C11—C12—N4	125.82 (14)	C27—C32—N8	116.71 (12)
C7—C12—N4	115.11 (12)	N8—C33—C34	122.69 (14)
N4—C13—C14	121.94 (14)	N8—C33—H33	120.2 (9)
N4—C13—H13	121.9 (10)	C34—C33—H33	117.1 (9)
C14—C13—H13	116.1 (10)	C35—C34—C39	118.27 (14)
C19—C14—C15	118.66 (14)	C35—C34—C33	119.59 (13)
C19—C14—C13	119.64 (14)	C39—C34—C33	122.11 (13)
C15—C14—C13	121.70 (13)	C36—C35—C34	120.77 (15)
C16—C15—C14	120.17 (14)	C36—C35—H35	118.7 (9)
C16—C15—H15	122.5 (9)	C34—C35—H35	120.5 (9)
C14—C15—H15	117.3 (9)	C35—C36—C37	121.15 (15)
C15—C16—C17	121.55 (15)	C35—C36—H36	119.8 (10)
C15—C16—H16	117.6 (10)	C37—C36—H36	119.0 (10)
C17—C16—H16	120.7 (10)	C36—C37—C38	117.97 (14)
C18—C17—C16	117.97 (14)	C36—C37—C40	121.10 (14)
C18—C17—C20	121.12 (15)	C38—C37—C40	120.91 (15)
C16—C17—C20	120.91 (15)	C39—C38—C37	121.34 (15)
C19—C18—C17	121.03 (15)	C39—C38—H38	119.0 (9)
C19—C18—H18	120.0 (10)	C37—C38—H38	119.7 (9)
C17—C18—H18	119.0 (10)	C38—C39—C34	120.47 (14)
C18—C19—C14	120.62 (15)	C38—C39—H39	120.9 (8)
C18—C19—H19	121.2 (10)	C34—C39—H39	118.6 (8)
C14—C19—H19	118.2 (10)	C37—C40—H40A	110.8 (10)
C17—C20—H20A	112.0 (14)	C37—C40—H40B	114.9 (11)
C17—C20—H20B	111.1 (12)	H40A—C40—H40B	104.2 (15)
H20A—C20—H20B	108.0 (19)	C37—C40—H40C	112.1 (10)
C17—C20—H20C	111.5 (14)	H40A—C40—H40C	107.4 (14)
H20A—C20—H20C	105.7 (19)	H40B—C40—H40C	106.9 (15)
H20B—C20—H20C	108.4 (18)	H3B—O3—H3C	106.3 (19)
C22—N5—N6	112.71 (12)		
			
C2—N1—N2—C4	−0.19 (15)	C22—N5—N6—C24	0.17 (15)
N2—N1—C2—C3	−0.09 (16)	N6—N5—C22—C23	0.04 (16)
N2—N1—C2—C1	−179.53 (13)	N6—N5—C22—C21	−179.52 (13)
N1—C2—C3—C4	0.31 (16)	N5—C22—C23—C24	−0.23 (16)
C1—C2—C3—C4	179.67 (15)	C21—C22—C23—C24	179.27 (16)
N1—N2—C4—C3	0.39 (15)	N5—N6—C24—C23	−0.32 (15)
N1—N2—C4—C5	−177.40 (12)	N5—N6—C24—C25	177.99 (12)
C2—C3—C4—N2	−0.45 (17)	C22—C23—C24—N6	0.35 (16)
C2—C3—C4—C5	177.10 (13)	C22—C23—C24—C25	−177.80 (14)
N2—C4—C5—C6	−120.07 (14)	N6—C24—C25—C26	75.24 (18)
C3—C4—C5—C6	62.6 (2)	C23—C24—C25—C26	−106.77 (17)
C7—N3—C6—O1	2.3 (2)	C27—N7—C26—O2	2.3 (2)
C7—N3—C6—C5	−178.85 (12)	C27—N7—C26—C25	−178.09 (13)
C4—C5—C6—O1	−154.35 (13)	C24—C25—C26—O2	167.33 (13)
C4—C5—C6—N3	26.74 (18)	C24—C25—C26—N7	−12.3 (2)
C6—N3—C7—C8	−4.5 (2)	C26—N7—C27—C28	12.5 (2)
C6—N3—C7—C12	174.38 (13)	C26—N7—C27—C32	−167.60 (13)
N3—C7—C8—C9	176.28 (14)	C32—C27—C28—C29	−2.3 (2)
C12—C7—C8—C9	−2.6 (2)	N7—C27—C28—C29	177.62 (13)
C7—C8—C9—C10	0.4 (3)	C27—C28—C29—C30	−0.8 (2)
C8—C9—C10—C11	1.6 (3)	C28—C29—C30—C31	2.0 (2)
C9—C10—C11—C12	−1.3 (3)	C29—C30—C31—C32	−0.1 (2)
C10—C11—C12—C7	−0.9 (3)	C30—C31—C32—C27	−2.9 (2)
C10—C11—C12—N4	−177.53 (16)	C30—C31—C32—N8	−179.62 (13)
C8—C7—C12—C11	2.8 (2)	C28—C27—C32—C31	4.1 (2)
N3—C7—C12—C11	−176.12 (13)	N7—C27—C32—C31	−175.82 (12)
C8—C7—C12—N4	179.83 (12)	C28—C27—C32—N8	−178.92 (12)
N3—C7—C12—N4	0.90 (18)	N7—C27—C32—N8	1.15 (18)
C13—N4—C12—C11	−6.3 (2)	C33—N8—C32—C31	−30.9 (2)
C13—N4—C12—C7	176.92 (13)	C33—N8—C32—C27	152.35 (13)
C12—N4—C13—C14	175.65 (13)	C32—N8—C33—C34	176.83 (12)
N4—C13—C14—C19	−177.01 (15)	N8—C33—C34—C35	−177.46 (14)
N4—C13—C14—C15	2.4 (2)	N8—C33—C34—C39	0.4 (2)
C19—C14—C15—C16	0.8 (2)	C39—C34—C35—C36	−0.7 (2)
C13—C14—C15—C16	−178.54 (14)	C33—C34—C35—C36	177.20 (14)
C14—C15—C16—C17	0.0 (2)	C34—C35—C36—C37	−0.2 (2)
C15—C16—C17—C18	−0.7 (2)	C35—C36—C37—C38	0.8 (2)
C15—C16—C17—C20	179.76 (16)	C35—C36—C37—C40	−177.95 (15)
C16—C17—C18—C19	0.6 (3)	C36—C37—C38—C39	−0.4 (2)
C20—C17—C18—C19	−179.91 (17)	C40—C37—C38—C39	178.34 (14)
C17—C18—C19—C14	0.3 (3)	C37—C38—C39—C34	−0.6 (2)
C15—C14—C19—C18	−1.0 (2)	C35—C34—C39—C38	1.1 (2)
C13—C14—C19—C18	178.41 (15)	C33—C34—C39—C38	−176.75 (13)

### Hydrogen-bond geometry (Å, º)

*Cg*1, *Cg*3 and *Cg*6 are the centroids of the N1/N2/C2–C4, C14–C19 and C34–C39 rings, respectively.

**Table:**

*D*—H···*A*	*D*—H	H···*A*	*D*···*A*	*D*—H···*A*
N1—H1···O2^i^	0.92 (2)	1.88 (2)	2.7863 (18)	169 (2)
O3—H3*B*···N2	0.92 (2)	1.91 (2)	2.8047 (19)	165 (2)
O3—H3*C*···N6^ii^	0.87 (2)	2.09 (2)	2.9530 (18)	174 (2)
N5—H5···O3^iii^	0.93 (2)	1.878 (19)	2.8014 (18)	173 (2)
N7—H7···N6	0.91 (2)	2.447 (17)	3.1314 (19)	132.6 (13)
C1—H1*A*···O1^iv^	0.99 (2)	2.56 (2)	3.436 (2)	146.9 (15)
C8—H8···O1	0.98 (2)	2.228 (15)	2.858 (2)	120.8 (12)
C28—H28···O2	1.01 (2)	2.265 (18)	2.890 (2)	118.5 (13)
C35—H35···N4^v^	0.99 (2)	2.532 (18)	3.451 (2)	155.2 (13)
N3—H3*A*···*Cg*1	0.91 (2)	2.999 (15)	3.6216 (17)	127.4 (12)
C5—H5*B*···*Cg*6^ii^	1.00 (2)	2.820 (16)	3.7171 (18)	149.0 (12)
C11—H11···*Cg*6	0.98 (2)	2.837 (19)	3.713 (2)	149.8 (14)
C15—H15···*Cg*1	0.99 (2)	2.913 (15)	3.7979 (19)	149.8 (12)
C20—H20*B*···*Cg*3^i^	1.00 (2)	2.88 (2)	3.772 (3)	148.9 (16)

Symmetry codes: (i) −*x*+1, −*y*+1, −*z*+1; (ii) *x*, *y*, *z*−1; (iii) −*x*, −*y*+1, −*z*+1; (iv) −*x*, −*y*, −*z*; (v) −*x*, −*y*, −*z*+1.
